# Patient-Derived Colorectal Cancer Extracellular Matrices Modulate Cancer Cell Stemness Markers

**DOI:** 10.3390/ijms26072890

**Published:** 2025-03-22

**Authors:** Ângela Marques-Magalhães, Sara Monteiro-Ferreira, Pedro Amoroso Canão, Elisabete Rios, Ângela Margarida Costa, Flávia Castro, Sérgia Velho, Joana Paredes, Fátima Carneiro, Maria José Oliveira, Ana Patrícia Cardoso

**Affiliations:** 1 i3S—Institute for Research and Innovation in Health, University of Porto, Rua Alfredo Allen 208, 4200-135 Porto, Portugal; angela.magalhaes@i3s.up.pt (Â.M.-M.); ferreiramonteirosara@gmail.com (S.M.-F.); culex.rios@gmail.com (E.R.); angela.amorimcosta@i3s.up.pt (Â.M.C.); flaviateixeiracastro@i3s.up.pt (F.C.); svelho@ipatimup.pt (S.V.); jparedes@ipatimup.pt (J.P.); fcarneiro@ipatimup.pt (F.C.); appcardoso@gmail.com (A.P.C.); 2 ICBAS—School of Medicine and Biomedical Sciences, University of Porto, Rua de Jorge Viterbo Ferreira 228, 4050-313 Porto, Portugal; 3 Champalimaud Research, Champalimaud Foundation, 1400-038 Lisbon, Portugal; 4 Centro Hospitalar Universitário São João, Alameda Professor Hernâni Monteiro, 4200-319 Porto, Portugal; pedro.a.canao@gmail.com; 5 FMUP—Faculty of Medicine of the University of Porto, Pathology Department, University of Porto, Alameda Professor Hernâni Monteiro, 4200-319 Porto, Portugal

**Keywords:** cancer stem cells, extracellular matrix, tissue decellularization, colorectal cancer

## Abstract

Although it has been shown that the tumor extracellular matrix (ECM) may sustain the cancer stem cell (CSC) niche, its role in the modulation of CSC properties remains poorly characterized. To elucidate this, paired tumor and adjacent normal mucosa, derived from colon cancer patients’ surgical resections, were decellularized and recellularized with two distinct colon cancer cells, HT-29 or HCT-15. Methods: The matrix impact on cancer stem cell marker expression was evaluated by flow cytometry and qRT-PCR, while transforming growth factor-β (TGF-β) secretion and matrix metalloprotease (MMP) activity were quantified by ELISA and zymography. Results: In contrast to their paired normal counterparts, the tumor decellularized matrices enhanced HT-29 expression of the pluripotency and stemness genes *NANOG* (*p* = 0.0117), *SOX2* (*p* = 0.0156), and *OCT4* (*p* = 0.0312) and of the epithelial-to-mesenchymal transition (EMT)-associated transcription factor *SNAI1* (*p* = 0.0156). Notably, no significant differences were found in the expression of *SLUG* or *TGFB* on HT-29 or of the six transcripts on HCT-15 cells. HT-29 mRNA alterations were followed by enhanced expression of the stemness-associated receptors cluster of differentiation 44 (CD44), CD133, and CD166 (*p* = 0.0078), the secretion of TGF-β (*p* = 0.0286), and MMP-2 (*p* = 0.0081) and MMP-9 (*p* = 0.0402) proteolysis. To infer the clinical relevance of these findings, we assessed cohort databases and evidenced that patients expressing higher levels of the four stemness-associated genes (*NANOG*/*SOX2*/*OCT4*/*SNAI1*) had worse overall survival. This study demonstrates that normal and tumor matrices harbor different stemness potential and suggest patient-derived decellularized matrices as an excellent three-dimensional (3D) model to unveil stemness signatures, appointing candidates for future therapeutic strategies.

## 1. Introduction

Colorectal cancer (CRC) is the second leading cause of cancer-related deaths [1], mainly due to the high rate of therapy resistance, disease relapse, and distant metastases [2]. Although being exploited, the mechanisms underlying this metastatic spread remain poorly understood. Under certain circumstances, cancer cells that spread before, or that remained after tumor resection and were resistant to therapy, are able to restore tumor growth. The interactions between these residual cancer cells, some of which have stemness properties, and the surrounding tumor microenvironment (TME) are decisive and can dictate the success of relapse [3].

Cancer stem cells (CSCs) are a subpopulation of cells within tumors with abilities of self-renewal, tumor initiation, and resistance to therapy [4]. By undergoing asymmetric division, CSCs can originate other stem cells or cancer cells without stem-like properties, giving rise to different populations that sustain tumor growth and its heterogeneity [4]. Generally, CSCs are more resistant to therapy and have been associated with the maintenance of minimal residual disease [5,6,7]. Nevertheless, their stemness potential can be modulated by other cellular and acellular elements of the TME, such as extracellular matrix (ECM) components [8].

The ECM is a dynamic macromolecular structure comprising core proteins, such as fibrillar and non-fibrillar collagens, glycoproteins (laminins, fibronectins), and proteoglycans, and by matrix-associated proteins, such as regulatory and secreted factors [9,10,11]. Depending on the tissue, different proportions of these elements establish a matrix network with distinct biochemical and biomechanical properties, providing cells mechanical support and acting as a reservoir of bioactive peptides and growth factors, tuning cell behavior [9,12]. Although it is known that the ECM may modulate stemness [4,8,12], the ability of normal or tumor matrices to differently impact this cancer hallmark remains unexplored.

Our hypothesis is that in contrast to the normal ECM, the tumor ECM can differently sustain the CSC niche, providing structural and biochemical support, regulating stem cell renewal and differentiation, and entrapping secreted growth factors, bioactive peptides and their receptors. To dissect the role of the ECM in cancer cell stemness, envisaging future therapeutic applications, a more reliable biomimetic system is required. In the past, most ECM studies were performed in two-dimensional (2D) culture systems, disregarding the spatial dimension and the cellular interactions established within the tumor microenvironment [13]. Although modern patient-derived three-dimensional (3D) models represent an improvement, they still do not faithfully mimic the chemical and biomechanical properties of both normal and diseased ECMs in the way that decellularized tissue models do. The decellularized (non-lyophilized) normal and tumor ECMs preserve the native biochemical composition and structural integrity more faithfully than other patient-derived 3D models, such as organoids, organotypic cultures, and bioprinting, where the ECM is either synthesized de novo, supplemented with a polymeric or reconstituted matrix, or structurally recreated. Thus, decellularized matrices represent an optimal model for studying ECM–tumor interactions [14,15]. Using human decellularized matrices, derived from CRC patients’ surgical resections, we previously demonstrated that tumor and paired normal adjacent mucosa retain native tissue features but differently modulate macrophage polarization [14]. In contrast to their normal counterparts, tumor decellularized matrices differentiated macrophages into a more anti-inflammatory profile, expressing low levels of the co-stimulatory CD86 receptor and high levels of CCL18. We further demonstrated that this immunosuppressive chemokine is abundant at the invasive front of more advanced CRC cases and has the ability to support cancer cell invasion [14].

In the present study, using paired tumor and normal decellularized matrices as bioactive scaffolds, derived from the same CRC patient surgical resection, we evaluated the impact of the ECM on the modulation of CSC-associated features and investigated the possible clinical relevance of the altered stemness signature.

## 2. Results and Discussion

### 2.1. Distribution Profiles of Colon Cancer Cells Differ on Normal vs. Tumor Matrices from Colorectal Cancer Patients

To dissect the role of the extracellular matrix, the most abundant component of the tumor microenvironment, on cancer stem cell properties, paired normal adjacent mucosa and tumor surgical resections, derived from the same colorectal cancer patient, were decellularized and characterized [14,16]. We have previously demonstrated via transversal to human and mouse tissues of distinct origin that the established protocol induces efficient tissue decellularization and extensive cell debris removal without traces of remaining DNA, while preserving major ECM components and their biomechanical properties [14].

**Figure 1:**
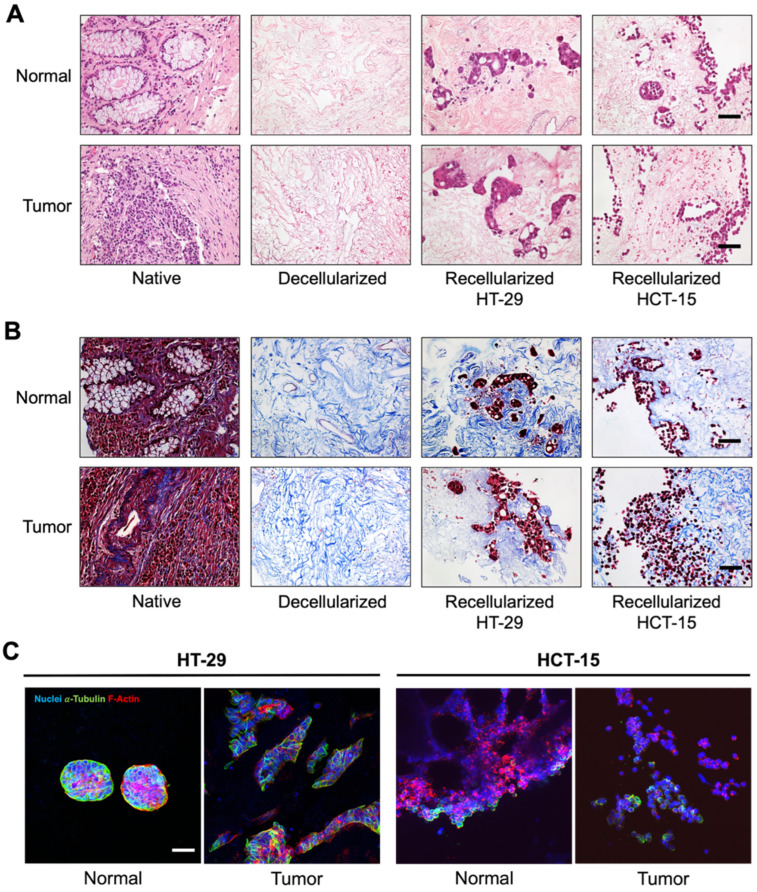
Decellularized human normal adjacent mucosa and tumor colorectal matrices, obtained from CRC patients’ surgical specimens, were recellularized with HT-29 or with HCT-15 colon cancer cells. Optical microscopy images of native, decellularized, and recellularized matrices. Hematoxylin and eosin (**A**) and Masson’s trichrome staining (**B**). Scale bar: 20 μm. (**C**) Confocal microscopy images of recellularized normal and tumor matrices. F-actin was stained with Alexa-Fluor-568-Phalloidin (red), α-tubulin with a monoclonal antibody following incubation with an Alexa-Fluor-488 secondary antibody (green), whereas nuclei were counterstained with DAPI (blue). Scale bar: 50 μm.

In the present study, hematoxylin–eosin (H&E) and Masson’s trichrome staining confirmed efficient decellularization with maintenance of the ECM meshwork (Figure 1A,B). Paired normal and tumor patient-derived matrices were then recellularized with 2.5 × 10^5^ HT-29 or 2.5 × 10^5^ HCT-15 colon cancer cells and maintained for 7 days in culture, at 37 °C in a 5% CO_2_ humidified atmosphere. Two colon cancer cell lines were used in this study.. The HT-29 cell line, derived from a microsatellite stable tumor (MSS) with a molecular profile of TP53^R273H^, KRAS^wt^, BRAF^V600E^, PIK3CA^wt^, PTEN^wt^, chromosomal instability (CIN^+^), and a CpG island methylator phenotype (CIMP^+^), with characteristics of consensus molecular subtype (CMS) 3 tumors, is frequently used in cell differentiation studies. The HCT-15 cell line, derived from a microsatellite instable tumor (MSI) with the molecular profile of TP53^S241F^, KRAS^G13D^, BRAF^wt^, PIK3CA^E545K^, PTEN^wt^, CIN^−^, and CIMP^+^, with characteristics of CMS1 tumors, is frequently used as it is representative of poorly differentiated tumors [17,18,19].

Recellularization was confirmed both by H&E and by Masson’s trichrome staining, evidencing the successful replenishment and distribution of cells within the ECM network (Figure 1A,B). Interestingly, across the distinct recellularized matrices, the cells revealed distinct infiltration patterns: the HT-29 cells aggregated into well-delineated clusters, while the HCT-15 cells displayed a more individual/single-cell colonization profile. Additional confocal microscopy images revealed that the HT-29 cells formed round-shaped agglomerates with well-defined cortical tubulin staining on normal matrices, in contrast with more elongated agglomerates with defined intercellular tubulin on tumor matrices. In turn, the HCT-15 cells displayed a diffused distribution pattern both on normal and on tumor ECMs. The pattern of HT-29 distribution on the tumor decellularized matrices was similar to that previously described by Parkinson et al. [20], a pioneer study that lacked the recellularization on normal matrices with other colon cancer cells. Of note, the different patterns observed may reflect the distinct architecture of normal and tumor matrices, as we previously evidenced [14], as well as the inherent properties of both cell lines, which may predispose to a different ECM distribution [17,18].

### 2.2. Tumor Matrices Induce HT-29 Colon Cancer Cells Stemness and Proteolysis

To uncover how normal and tumor matrices may differently impact the CSC molecular signature, gene expression analysis concerning a relevant pluripotency-associated stem cell marker complex *NANOG*/*SOX2*/*OCT4* [21] was performed. *NANOG* is a transcription factor that can sustain the pluripotency of human embryonic stem cells and is considered a key CSC marker [21,22], particularly in CRC, while SOX2 and OCT4 are involved in pluripotency regulation, de-differentiation, and epithelial-to-mesenchymal transition (EMT) in several gastrointestinal tumors [21,23].

**Figure 2:**
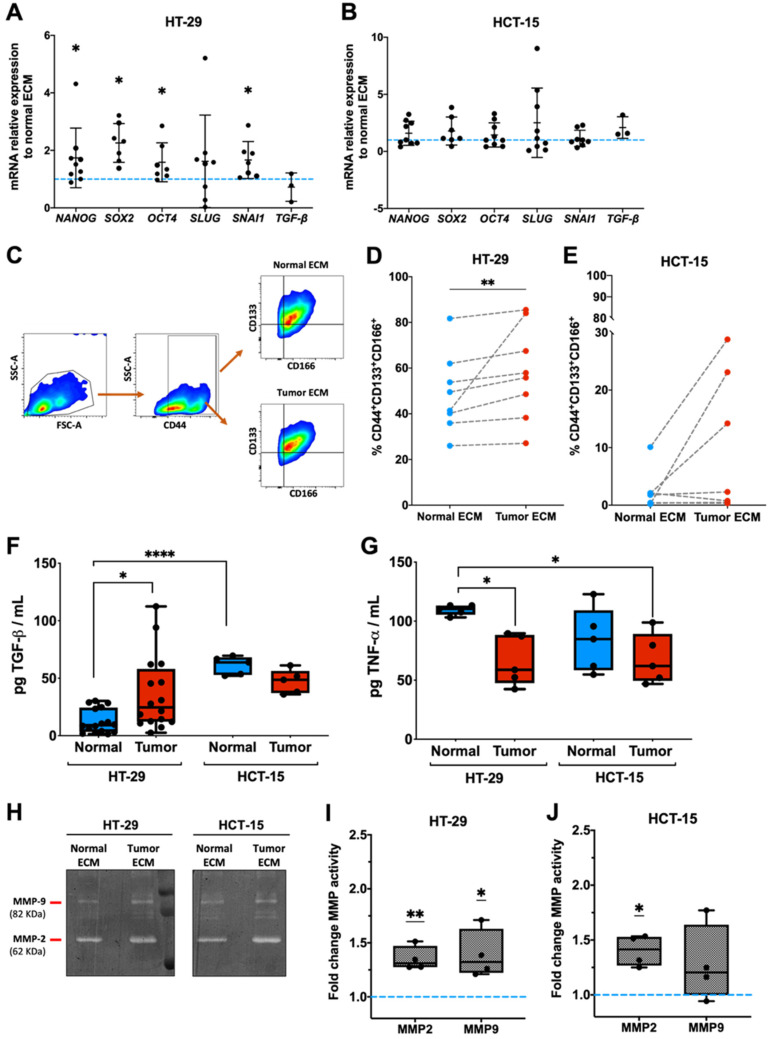
Tumor matrices drive the expression of CSC markers on HT-29 cells in comparison with their paired normal matrices. Gene expression fingerprint of HT-29 (**A**) and HCT-15 cells (**B**) (determined using qPCR) after growing for 7 days on tumor or paired normal decellularized matrices. Data are expressed relative to expression levels of paired normal matrices. *GAPDH* was used as housekeeping gene. Grey bars indicate mean ± SD. Dots indicate individual patients. * *p* < 0.05, Wilcoxon signed rank test was used to compare the median of each dataset against a hypothetical median value of 1 (dotted blue line). (**C**) Gating strategy for flow cytometry analysis of HT-29 and of HCT-15 cells after growing for 7 days on tumor or paired normal decellularized matrices. CD44^+^ cells were gated on single cells. CD166^+^CD133^+^, CD166^+^CD133^−^, CD166^−^CD133^+^, and CD166^−^CD133^−^ cells were gated on CD44^+^ cells. (**D**) The percentage of triple-positive HT-29 and HCT-15 cells (**E**) was determined. Dots represent values obtained with paired matrices from 8 (HT-29) or from 7 CRC patients (HCT-15), with the last being included in the first. ** *p* < 0.01; Wilcoxon matched-pairs signed rank test. TGF-β (**F**) and TNF-α (**G**) expression levels present in conditioned media from HT-29 and HCT-15 recellularized matrices were quantified by ELISA. Bars represent mean values obtained from paired normal or tumor matrices derived from 16 (HT-29) or from 15 (HCT-15) out of these 16 patients. * *p* < 0.05, **** *p* < 0.0001; Kruskal–Wallis test. Conditioned media were also run on gelatin zymograms. (**H**) Proteolytic activity bands were revealed in white gelatinolytic areas on a blue background stained with Coomassie. Densitometry analysis using QuantityOne^®^ software (Version 4.6.6, BioRad, Hercules, CA, USA) allowed quantification of MMP-9 and of MMP-2 activities in conditioned media from HT-29 (**I**) or HCT-15 recellularized matrices (**J**). * *p* < 0.05; ** *p* < 0.01; one sample t-test (normality according to Shapiro–Wilk test). MMP data correspond to mean values of independent experiments performed with matrices from 5 different patients. Bars indicate minimum and maximum values, and lines are the mean. Blue dotted line at Y = 1 represents normal ECM values. Statistical analysis was performed in GraphPad Prism 9.

Our results evidenced that the *NANOG* (*p* = 0.0117), *SOX2* (*p* = 0.0156), and *OCT4* (*p* = 0.0312) gene expression was significantly increased in HT-29 cells growing on tumor matrices when compared to those growing on their normal counterparts (Figure 2A). Similarly, the expression of *SNAI1*, a transcription factor with a fundamental role in EMT and associated with stemness properties [24], also exhibited the same behavior (*p* = 0.0156) (Figure 2A). Recently, Parkinson et al. demonstrated that patients’ scaffolds, obtained after tumor resection decellularization, also induced, in comparison to 2D cultures, alterations in the HT-29 expression profile with decreased expression of proliferation and increased expression of stemness and EMT markers [20]. Our study is, however, the first to consistently compare the impact of tumor versus matched normal matrices derived from the same CRC patient on colon cancer cell stemness. Notably, no significant differences were found in the expression of *SLUG* or of *TGFB*, which are frequently involved in cancer cell stemness, on HT-29 cells or on the six transcripts on HCT-15 cells (Figure 2B).

Based on recent studies on CRC stemness, the impact of both normal and tumor matrices on the expression of the stemness-associated cell surface receptors CD44, CD133, and CD166 was also investigated [25,26,27] (Figure 2C–E). Importantly, tumor matrices induced a significant enrichment of a CD44/CD133/CD166 triple-positive HT-29 sub-population (*p* = 0.0078), when compared to paired normal matrices (Figure 2D). Importantly, CD44 is simultaneously a stemness marker and the receptor for hyaluronic acid (HA), an abundant component of the tumor ECM [28] not affected by our decellularization protocol [14]. Previous studies demonstrated that stiffer matrices, which are generally those in tumors, enhanced breast cancer CD44 or colon cancer CD133 expression, contributing to the sustaining of cancer cell stemness [28,29,30]. Again, although some heterogeneity was observed, the tumor ECM did not have an impact on the HCT-15 cell stemness marker surface expression (Figure 2E). In fact, a previous study evidenced that the maintenance of HCT-15 stemness potential required the downregulation of miR-203 by HA/CD44 signaling [31]. The fact that these two cell lines have distinct underlying characteristics associated with differentiation in 2D, with HCT-15 being more undifferentiated, might also explain the higher responsiveness of HT-29 cells to the tumor ECM. Indeed, the basal expression levels of CRC stemness-associated genes are already higher in HCT-15 than in HT-29 cells [17], and the undifferentiated status of HCT-15 appears to impair its modulation by either the normal or the tumor ECM (Figure 2C–E).

In several studies, dysregulation of TGF-β signaling has been also associated with the CRC stem-like features of [32] and HCT-15 cells, as more undifferentiated cells are described as exhibiting increased expression of TGF-β-induced genes, which are already in standard 2D culture conditions [17]. In vivo, secreted latent forms of TGF-β are often arrested and stored within the complex ECM network and proteolytically cleaved and activated under certain anti-inflammatory and pro-fibrotic conditions [33]. In our system, *TGFB* was similarly expressed when cells were grown over normal or tumor matrices (Figure 2A,B). However, the TGF-β protein levels were significantly higher in the HT-29 recellularized tumor matrices than in their normal counterparts (*p* = 0.0286), suggesting that tumor matrices may promote its proteolytic activation (Figure 2F). Interestingly, TGF-β levels in HCT-15 recellularized normal matrices were significantly higher than in HT-29 recellularized normal matrices from the same patient, further substantiating the fact that variations between the normal and tumor ECM are less evident due to the high basal state of undifferentiation of HCT-15 cells. On the other hand, a decrease in tumor necrosis factor (TNF-α) secretion levels (*p* = 0.0382) was depicted in the tumor ECM compared with their paired normal counterparts for HT-29 cells (Figure 2G). Although many reports described the ability of this molecule to induce a stem-like phenotype [34,35], TNF-α is a double-edged sword for tumors, being involved in both cell death and survival, depending on the biologic context [36]. In CRC, the fact that this cytokine associates with cell death pathways might indicate that tumor cells cultured over a tumor ECM secreting less TNF-α than those in a normal ECM are probably dying less [37]. Additionally, the opposite secretion of both growth factors might be related to the inhibitory effect of TNF-α over TGF-β, as well as an impairment of ECM production, as previously described [38]. The decrease in TNF-α levels on HCT-29 recellularized tumor matrices favors the TGF-β effect, whose levels are increased. Therefore, in our model, the decreased secretion of TNF-α, accompanied by the increased expression of TGF-β might be potentiating the HT-29 stem-like phenotype, in contrast to HCT-15, where no differences were found.

ECM-arrested latent TGF-β can be activated through oxidative modifications, binding to integrins, interaction with the matrix protein Thrombospondin-1 (TSP-1), or through proteolysis, by the action of enzymes, as matrix metalloproteases (MMPs) [32,39]. Considering the enhanced TGF-β levels secreted by HT-29 cells differentiated on tumor matrices, we decided to analyze proteolysis, via gelatin zymography (Figure 2H–J). Thus, we confirmed that both MMP-2 (*p* = 0.0081) and MMP-9 (*p* = 0.0402) activities were significantly more enhanced in media collected from the tumor than from paired normal matrices recellularized with HT-29 cells (Figure 2I). In contrast, on HCT-15 recellularized matrices, only MMP-2 proteolytic activity was significantly enhanced (*p* = 0.0107) (Figure 2J). These data correlate with the disparities obtained regarding TGF-β levels, suggesting that MMPs might be involved in the proteolytic activation and release of this growth factor from the ECM. However, we cannot conclude whether the differences are due to distinct amounts of TGF-β present within normal and tumor matrices or due to the higher MMP activity, or other factors, induced by tumor matrices. Still, higher TGF-β levels in HT-29 recellularized tumor matrices could be responsible, at least partially, for the observed increase in stemness markers, since this growth factor is generally associated with the expression of *SNAI1* [40], *CD133* [41], *CD166* [42], *NANOG*, *OCT4*, and *SOX2* [43]. It would be interesting to use, in the near future, integrin-blocking assays and TGF-β, Lysyl oxidase (LOX), or YAP/TAZ inhibitors to dissect the molecular mechanisms associated with tumor matrix-induced stemness.

Overall, differences in inducing a stemness profile by tumor matrices compared with their normal counterpart were found only on HT-29 cells. The absence of this phenotype on HCT-15 might be associated with the intrinsic characteristics of the cell line, namely microsatellite repair status, mutations, and CMS. In fact, a recent study demonstrated that MSI-H and CMS1 tumors tended to be characterized by a reduced stemness profile, while the mesenchymal subtype CMS4 was the prevalent of high stemness features [43]. Interestingly, in addition to the alterations already described [18], HT-29 cells do not harbor mutations on TGF-β receptor genes, while HCT-15 cells exhibit *TGFBR3* mutations, according to the catalogue of somatic mutations in cancer (COSMIC). This may underline the mechanisms behind the different responses of HT-29 and HCT-15 cells to the extracellular matrix and should be further exploited.

### 2.3. The Stemness Signature Triggered by Tumor Decellularized Matrices Is Associated with Patients’ Worse Overall Survival

**Figure 3:**
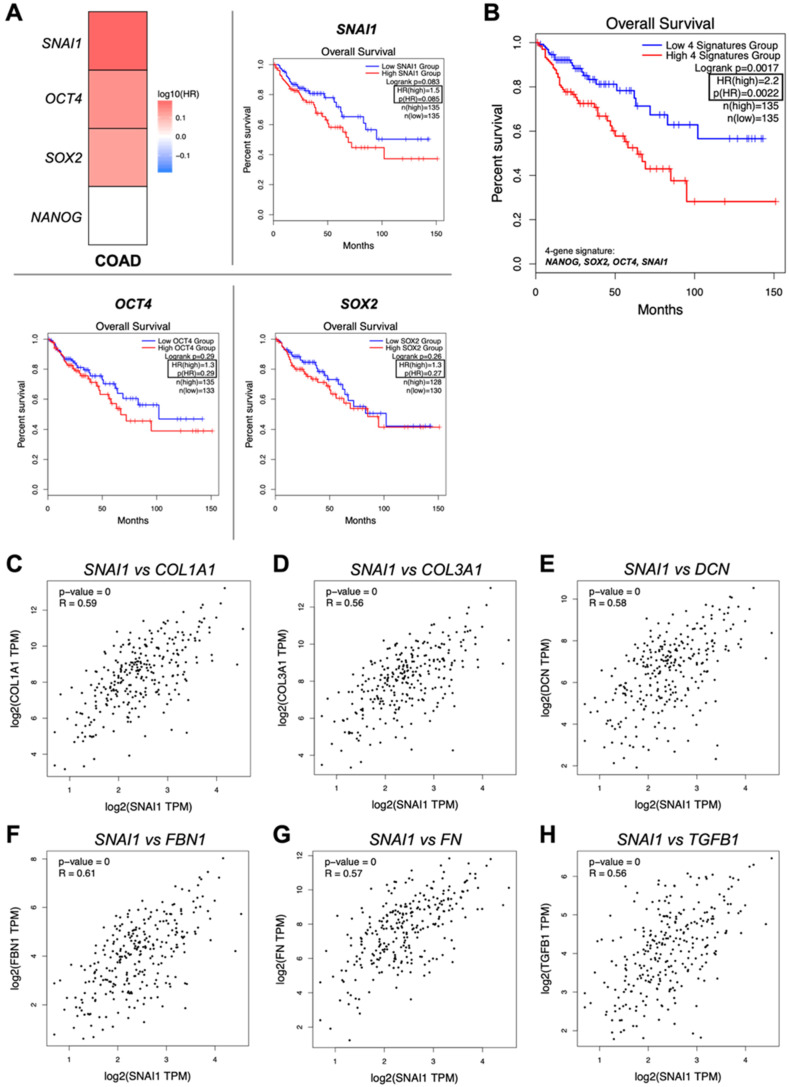
Stemness-associated gene signature correlates with worse overall survival (OS) and ECM-associated genes. Relationship between the four-gene signature (*NANOG*/*SOX2*/*OCT4*/*SNAI1*) and OS of patients in the publicly available TCGA-COAD dataset from GEPIA 2 (http://gepia2.cancer-pku.cn/#index, accessed on 2 September 2024). Survival map and Kaplan–Meier curves of COAD patients’ cohort for each stemness-associated gene (**A**) and as a four-gene signature (**B**). The COAD patient cohort was divided into two groups (low and high four-gene signature, n = 135 each group), defined based on the median value. The log-rank *p*-value (*p* = 0.0017) and hazards ratio (HR) of the Kaplan–Meier curve comparison between the high (red) and low (blue) expression groups is shown. Pearson correlation between *SNAI1* and ECM-associated genes, namely *COL1A1* (**C**), *COL1A3* (**D**), *DCN* (**E**), *FBN1* (**F**), *FN* (**G**), and *TGFB1* (**H**). Correlation coefficients and *p*-values are shown.

Analysis of a colon adenocarcinoma (COAD) patient cohort, from The Cancer Genome Atlas (TCGA), revealed that the transcript levels of the individual stemness-associated genes, upregulated in HT-29 recellularized tumor matrices (*NANOG*, *SOX2*, *OCT4*, and *SNAI1*), have no impact on patients’ overall survival (OS) (Figure 3A). However, patient groups expressing high levels of *SNAI1* tend to have a worse OS than those patients expressing low levels of this stemness-associated gene (Hazards Ratio (HR) = 1.5, *p* = 0.085). Importantly, when considering the four stemness-associated gene signatures (*NANOG*, *SOX2*, *OCT4*, and *SNAI1*), differently expressed by HT-29 cells on tumor matrices, a clearly decrease in overall survival was observed for the high-expression group of patients (Figure 3B). In fact, the rate of deaths in this group is twice the rate of the low-expression group (HR = 2.2, *p* = 0.0022). These results suggest that the stem-like phenotype is associated with a worse patient overall survival. An educated-guess approach, based on a limited number of ECM proteins (n = 33) capable of inducing stem properties, namely collagens, proteoglycans, glycoproteins (laminins, fibronectins, and others), and secreted factors [8], revealed a positive correlation (Pearson coefficient (R) = 0.56–0.61) between *SNAI1* gene expression and *COL1A1*, *COL1A3*, *DCN*, *FBN1*, *FN*, and *TGFB1* (Figure 3C–H). However, no correlations were found between *NANOG*, *SOX2*, and *OCT4* expression and the list of ECM proteins considered.

**Figure 4:**
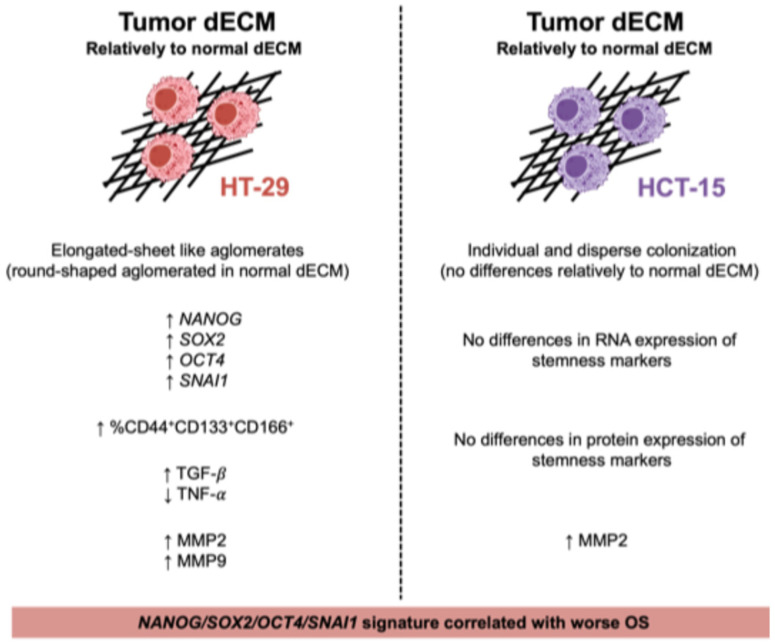
Patient-derived decellularized matrices differently impact colon cancer cell behavior. Decellularized tumor matrices induced HT-29 elongated sheet-like agglomerates with enhanced expression of *NANOG*/*SOX2*/*OCT4*/*SNAI1*, increased expression of CD44^+^CD133^+^CD66^+^ cells, increased TGF-β, decreased TNF-α, and enhanced MMP-2 and MMP-9 activities. In contrast, decellularized tumor matrices induced HCT-15 cells’ individual and dispersed colonization with no differences in RNA or protein expression of stemness markers but with enhanced MMP-2 activity. Arrows indicate the direction of change: ↑ represents an increase, while ↓ denotes a decrease.

The groundbreaking proteomics study of Naba et al. [44] on decellularized human colon matrices, revealed that these six ECM proteins (COL1A1, COL1A3, DCN, FBN1, FN, and TGFB) are present in the tumor matrisome. This finding appoints them as promising candidates for future research as potential inducers of stemness features on cancer cells. Specifically, type I collagen (COL1A1), the most abundant ECM protein, is described as inhibiting the differentiation and as promoting a stem cell-like phenotype in human CRC cells [45]. Additionally, increased MMP-2 secretion by cancer cells might indicate a high content of COL1A1, since this enzyme preferentially cleaves this type of collagen. Importantly, as we have previously shown that tumor decellularized matrices are stiffer than their normal counterparts, the biomechanical properties of the ECM should also be considered in future studies since it has been reported that ECM stiffness modulates cancer cell stemness [28,46]. Although others have already demonstrated the potential of decellularized matrix models in inducing breast cancer stemness properties [47,48], this corresponds, according to our knowledge, to the first study demonstrating the stemness potential of patient-derived colon tumor matrices and evidences the association of the stemness signature found with patients’ overall survival (Figure 4).

## 3. Materials and Methods

### 3.1. Clinical Samples and Ethics Statement

Fresh surgical specimens, derived from CRC patients, were kindly provided by the Pathology Department from Centro Hospitalar Universitário São João (CHUSJ). Paired tumor and normal adjacent mucosa were transported in cold Hank’s Balanced Salt Solution Medium (HBSS) (Gibco, Invitrogen, Waltham, MA, USA) to i3S and cut into smaller fragments to be cryopreserved. Tissue fragments were immediately immersed in mounting medium, immediately frozen in 2-methylbutane (VWR), and stored at −80 °C until further use. Patient-derived matrices were assessed through a protocol established between i3S and CHUSJ and approved by the hospital ethics committee (259/11 and 260/11) and used upon signed informed consent agreement, obtained prior to collection.

### 3.2. Cell Lines and Culture Conditions

Two human CRC cell lines were used: the MSI HCT-15 (CCL-225™) and the MSS HT-29 (HTB-38™), derived from primary colon adenocarcinomas and purchased from the American Type Culture Collection (ATCC, Manassas, VA, USA). The cells were genotyped and cultured in RPMI1640 medium (Invitrogen) supplemented with 1% penicillin-streptomycin (Pen/Strep) (Invitrogen) and 10% fetal bovine serum (FBS) (Biowest, Bradenton, FL, USA). The cells were maintained at 37 °C in a 5% CO_2_ humidified atmosphere.

### 3.3. Tissue Decellularization

Normal and tumor tissues, derived from CRC patients’ surgical resections, were exposed to a decellularization protocol, optimized by our group [14], to remove all cellular components, while maintaining tissue architecture and biomechanical properties. Thus, normal and tumor frozen colorectal specimens, from the same patient, were washed in phosphate-buffered saline (PBS), cut into similar cubes using a 4 mm biopsy disposable punch, and placed in a 24-well plate. The decellularization protocol lasted 3 days and involved chemical, enzymatic, and physical processes. The protocol was performed under agitation at 165 rpm and at room temperature (RT). Briefly, fragments were incubated in hypotonic buffer A (10 mM Tris; 0.1% EDTA pH 7.8) for 18 h, washed three times in PBS (1 h/wash) and incubated with 0.2% sodium dodecyl sulfate (SDS) for 24 h. Fragments were washed three times with hypotonic buffer B (10 mM Tris, pH 7.8) (20 min/wash) and incubated for 3 h with a DNAse solution (50 U/mL DNAse (Applichem, Darmstadt, Germany) in 100 mM Tris, 20 mM MgCl_2_ pH 7.8) at 37 °C. Three final washes with PBS were performed (1 h/wash) to remove residual detergent or DNAse. Decellularized matrices were kept at 4 °C in PBS with gentamicin (10 μg/mL) and 1% fungizone (Gibco) for a maximum of 7 days. Matrices were used as scaffolds for cell repopulation studies or fixed, using formalin, for characterization studies.

### 3.4. Tissue Recellularization

The matrices were washed with PBS twice and incubated with RPMI1640 supplemented with 1% Pen/Strep (Biowest) and 10% FBS (Biowest), for at least 30 min. Normal and tumor matrices were transferred to a 96-well plate with a U-bottom. Then, 25 × 10^4^ HCT-15 or HT-29 cells were resuspended in 10 μL of culture medium RPMI1640 (Invitrogen) supplemented with 1% Pen/Strep (Invitrogen) and 10% FBS (Biowest), placed on top of each matrix, and incubated for 18 h at 37 °C in a humidified 5% CO_2_ atmosphere. The matrices were then transferred to new wells, maintained for 7 days, while 200 μL of culture medium was changed every 2 days. Conditioned media were collected for further analysis, and cells were detached using Accutase (GRISP, Porto, Portugal). The activity of this enzyme was inhibited, as previously described, and cells were counted with a Burker chamber and further used.

### 3.5. Hematoxylin and Eosin Staining

Decellularized normal and tumor matrices were fixed using formalin with 0.02% eosin, embedded in paraffin, and cut into 3 μm thick sections to perform H&E staining. Briefly, the slides were sequentially immersed in Clear-Rite (Thermo, Gosselies, Belgium) for 15 min (twice), 100% ethanol for 5 min (twice), 70% ethanol for 5 min, tap water for 5 min, Hematoxylin Gill II (Thermo) for 2 min, running tap water for 6 min, 95% ethanol for 1 min, eosin (Thermo) for 4 min, 95% ethanol for 1 min, 100% ethanol for 2 min (twice) and Clear-Rite (Thermo) for 2 min. The slides were then mounted with Richard-Allan Scientific Mounting Medium (Thermo) and left to dry overnight in a fume hood and visualized using a fluorescent microscope (Axiovert 200 M, Zeiss, Oberkochen, Germany).

### 3.6. Masson’s Trichrome Staining

Reagents were from the Masson’s trichrome kit (Sigma), except for the Bouin’s solution and the mounting medium. The slides were sequentially immersed in Clear-Rite for 15 min (twice), 100% ethanol for 5 min (twice), 70% ethanol for 5 min, tap water for 5 min, Bouin’s solution (VWR, Belgium) for 2 h, running tap water until removal of yellow color, deionized water for 2 min, working iron hematoxylin solution for 10 min, running tap water for 10 min, deionized water for 2 min, Biebrich Scarlet-Acid Fuchsin solution for 5 min, and deionized water for 2 min. The slides were placed in phosphomolybdic-phosphotungstic acid solution for 5–10 min, immersed in aniline blue solution for 5 min, washed in distilled water, and placed in 1% acetic acid solution for 3–5 min. Slide dehydration was performed by immersion in 95% ethanol for 3 min (twice), 100% ethanol for 3 min (twice) and Clear-Rite for 5 min (thrice). The slides were then mounted with Richard-Allan Scientific Mounting Medium (Fisher, Waltham, MA, USA) and dried overnight in a fume hood; prior visualization was conducted using an Axiovert 200 M inverted fluorescent microscope (Zeiss).

### 3.7. DAPI Staining

The slides were sequentially immersed in Clear-Rite for 10 min (twice), 100% ethanol for 5 min, 70% ethanol for 5 min, washed with deionized water for 5 min (twice), and mounted with Vectashield containing DAPI (Vectashield, Burlingame, CA, USA). The slides were dried overnight and visualized with the Axiovert 200 M inverted fluorescent microscope (Zeiss).

### 3.8. Flow Cytometry

To evaluate the impact of the matrices on distinct stem cell markers, the expression of the cell surface receptors CD133, CD166, CD44, CD24, CD49f, and PD-L1 were analyzed by flow cytometry. Thus, detached HCT-15 or HT-29 cells were centrifuged at 1500 rpm for 5 min at 4 °C. Pellets were resuspended in flow cytometry buffer (PBS, 2% FBS, 0.01% sodium azide) and stained with PE anti-human CD166 (BioLegend, San Diego, CA, USA), FITC anti-human CD44 (BioLegend), APC anti-human CD133 (BioLegend), FITC anti-human PD-L1 (BD Biosciences, Franklin Lakes, NJ, USA), PE-Cy5 anti-human CD49f (BD Biosciences), and PE anti-human CD24 (Miltenyi Biotec, Bergisch Gladbach, Rhineland, Germany). Mouse IgG1 control PE, mouse IgG1 control FITC, and mouse IgG1 control APC (all ImmunoTools, Friesoythe, Germany) were used as isotypic controls. After additional washes with flow cytometry buffer, the cells were fixed with 1% paraformaldehyde (PFA) for 10 min at 4 °C. After 3 additional washes, the samples were filtered, and the cells were acquired on a Canto flow cytometer (BD Biosciences) using FACS Diva software (v6.1.3). Data analysis was performed with FlowJo software (v10).

### 3.9. Enzyme-Linked Immunosorbent Assay (ELISA)

The levels of TNF-α and TGF-β, in conditioned media from HCT-15 or HT-29 repopulated matrices, were measured through enzyme-linked immunosorbent assay (ELISA) (BioLegend), according to the manufacturer’s instructions. Briefly, samples and standards were incubated for 2 h in 96-well plates with a capture antibody specific to each cytokine. After washing, a detection antibody, specific for the cytokine of interest, was added to the wells and incubated for 1 h with agitation. Then, the wells were washed, incubated with Avidin-HRP D solution for 30 min with agitation, washed, and incubated with substrate solution F for 10 min in the dark, followed by the addition of the stop solution. The absorbance was measured in a synergy fluorescence microplate reader (BioTek, Winooski, VT, USA) and correlated with the cytokine concentration (pg/mL).

### 3.10. RNA Extraction

To evaluate the expression of several pluripotency and epithelial-to-mesenchymal transition (EMT)-related genes, total RNA was extracted from cells previously cultured on normal or tumor matrices. Detached cells were centrifuged at 1200 rpm for 5 min at 4 °C, resuspended in 500 μL of TriPure Isolation Reagent (Roche, Basel Switzerland) and transferred into 1.5 mL Eppendorf tubes. The cells were then stored at −80 °C until further use. For RNA extraction, the cells were incubated for 5 min at RT to ensure complete dissociation of nucleoprotein complexes. After incubation, 100 μL of chloroform was added, shaken vigorously for approximately 15 s, incubated at RT for 15 min, and centrifuged at 12,000× *g* for 15 min at 4 °C, to separate the solution into three phases. Afterwards, the colorless upper part was transferred into new tubes. To precipitate the RNA, isopropanol was added (250 μL/tube) and gently inverted, followed by an incubation at RT for 10 min. Afterwards, the tubes were centrifuged at 12,000× *g* for 10 min at 4 °C, and the supernatant was discarded. Ethanol was added (500 μL/tube), and the RNA was washed by vortexing; then, it was centrifuged at 7500× *g* for 5 min at 4 °C. The supernatant was discarded, and the excess ethanol was removed by air drying for 1 h. Subsequently, each pellet was resuspended in 20 μL of RNAse-free water and incubated for 1 h, at 4 °C. The RNA concentration and purity were analyzed by Nanodrop (Thermo) and stored at −80 °C until further use.

### 3.11. Quantitative Real-Time PCR

After cDNA synthesis, qRT-PCR was carried out using a TaqMan Universal PCR Master Mix (KAPA PROBE FAST qPCR Master Mix, KAPA Biosystems, Wilmington, MA, USA) and probes for *NANOG* (*Hs.PT.58.21480849*), *SNAI1* (*Hs.PT.58.2984401*), *SNAI2* (*Hs.PT.58.1772559*), *ITGA6A* (*Hs.PT.58.71647180*), *ITGA6Β* (*Hs.PT.58.71647184*), *VIM* (*Hs.PT.58.38906895*), *FN1* (*Hs.PT.58.40005963*), *SOX2* (*Hs.PT.58.237897.g*), *OCT4/POU5F1* (*Hs.PT.58.14648152.g*), *ZEB1* (*Hs.PT.58.39178574*), *TGFΒ1* (*Hs.PT.58.39813975*), and *GAPDH* (*Hs.PT.39a.22214836*) (housekeeping gene). The qRT-PCR was performed in a 7500 Real Time PCR system (Applied Biosystems, Waltham, MA, USA). The program was 95 °C for 20 s followed by 40 cycles of 95 °C for 3 s and 60 °C for 30 s. Reactions were conducted in triplicate, and the relative mRNA expression of the target genes was normalized to the levels of the housekeeping gene (*GAPDH*), using the comparative ΔΔCT method.

### 3.12. Gelatin Zymography

To evaluate matrix metalloprotease (MMP) activity, conditioned media from normal or tumor matrices, repopulated with HCT-15 and HT-29, were analyzed by gelatin zymography. Media were collected and centrifuged at 1300 rpm for 3 min at 4 °C to eliminate cellular debris, while supernatants were transferred to a new Eppendorf tube. The protein concentration was determined through the DC protein assay kit (BioRad), and 15 μg of protein were mixed with a non-reducing sample buffer (10% SDS, 4% sucrose, and 0.03% bromophenol blue in 0.5 M Tris-HCl pH 6.8) and PBS. Separating (10% polyacrylamide, 0.1% gelatin (bovine skin type B, Sigma G9391) and stacking (2.5% polyacrylamide) gels were prepared. The samples were loaded and separated for approximately 4 h at 80 V. After electrophoresis, the gels were washed twice with 2% Triton X-100 and incubated for 16 h at 37 °C in MMP substrate buffer (50 mM Tris-HCl; pH 7.5, 10 mM CaCl_2_, and 0.5% NaN_3_). Afterwards, the gels were stained with Coomassie blue solution (Sigma Aldrich, St. Louis, MO, USA) for 30 min, followed by deionized water wash. White bands of proteolytic degradation appeared on a blue background. Finally, the gels were scanned, and the MMPs’ molecular weight and activity were estimated by densitometric analysis (QuantityOne, BioRad).

### 3.13. Statistical Analysis

All analyses and graphing were performed using the GraphPad Prism Software 6.0 (GraphPad-trial version). Data were analyzed for Gaussian distribution using D’Agostino–Pearson or Shapiro–Wilk normality tests. For non-parametric samples, Mann–Whitney tests were used, while for parametric samples, t-tests were applied. The experiments were treated as means ± SD. Statistical significance was achieved at a *p* value < 0.05.

## 4. Conclusions

This study highlights the unknown role of the tumor ECM to maintain the colon CSC niche and the importance of dissecting the associated molecular signatures to unveil their impact on CRC patients’ overall survival. The future identification of the biochemical and/or biomechanical elements responsible for these interactions will be paramount to disclose the mechanisms that modulate cell stemness/differentiation and provide potential targets for future therapeutic intervention.

## Data Availability

The original contributions presented in this study are included in the article. Further inquiries can be directed to the corresponding author.

## Abbreviations

CSC	Cancer stem cell
CRC	Colorectal Cancer
ECM	Extracellular matrix
